# A Randomized Clinical Trial of a Standardized Pumpkin-Seed-Derived Peptide Extract: Body Composition Outcomes and Exploratory Analysis by Baseline Adiposity in Overweight Adults

**DOI:** 10.3390/ijms27198813

**Published:** 2026-10-01

**Authors:** Yu-Cheng Lin, Wan-Yu Chung, Yen-Nung Lin, Pang-Kuei Hsu

**Affiliations:** 1 Graduate Institute of Food Science and Technology, National Taiwan University, Taipei 10617, Taiwan; d08641002@ntu.edu.tw; 2 Research and Development Department, Greenyn Biotechnology Co., Ltd., Taichung City 40768, Taiwan; 3 Department of Physical Medicine and Rehabilitation, Wan-Fang Hospital, Taipei Medical University, Taipei 11696, Taiwan

**Keywords:** pumpkin seed peptides, bioactive peptides, body composition, baseline adiposity, dual-energy X-ray absorptiometry, treatment response heterogeneity, overweight adults

## Abstract

This 12-week, randomized, double-blind, placebo-controlled trial enrolled 50 overweight adults who received either a standardized pumpkin-seed-derived preparation (IRK-19^®^, 600 mg/day) or placebo. The clinical preparation contained 66.2% crude protein; components with apparent molecular weights ≤6.29 kDa accounted for 23.6% of the total SEC-UV chromatographic area, and the combined HPLC-measured content of six selected peptide markers was 2.79% (*w*/*w*). Among the 48 participants with evaluable DXA data at Week 12, between-group comparisons of body weight, BMI, and adiposity-related outcomes did not reach statistical significance in the overall study population. In a post hoc exploratory subgroup defined by baseline total fat mass ≥28.4 kg (the study-specific 75th percentile; *n* = 13), adjusted between-group differences (IRK-19^®^ minus placebo) at Week 12 were −2.30 kg for body weight (95% CI, −4.08 to −0.51; *p* = 0.018), −0.75 kg/m^2^ for BMI (95% CI, −1.48 to −0.02; *p* = 0.045), and −1.60 kg for total fat mass (95% CI, −3.23 to 0.04; *p* = 0.055). Treatment-by-baseline-fat-mass interactions did not reach statistical significance for body weight, BMI, or total fat mass (*p* = 0.065, 0.106, and 0.057, respectively). These post hoc subgroup findings warrant further investigation but were not adjusted for multiple comparisons. Prospectively designed trials with prespecified adiposity strata are needed to determine whether baseline fat mass is associated with differential treatment response. Individual metabolic laboratory abnormalities also warrant further assessment in future prospective trials.

## 1. Introduction

Overweight and obesity are commonly classified using body mass index (BMI), yet BMI does not directly reflect the amount, distribution, or physiological consequences of adipose tissue. Contemporary obesity frameworks have therefore increasingly emphasized assessment of excess adiposity and obesity-related organ or metabolic dysfunction rather than reliance on body size alone [1,2]. This distinction is clinically relevant because individuals with similar BMI values may differ substantially in total fat burden, regional fat distribution, and adipose tissue function. Adipose tissue is not merely an energy storage compartment but a metabolically active organ whose expandability, cellular characteristics, and anatomical distribution can influence systemic metabolic health [3]. These considerations support the use of body compositionbody composition measures alongside conventional anthropometric indices when evaluating interventions targeting adiposity or weight-related outcomes.

Responses to nutritional and weight management interventions are also highly heterogeneous. Individuals receiving the same intervention may exhibit substantially different changes in body weight, adiposity, and metabolic outcomes, reflecting differences in baseline physiology, behavior, and other individual characteristics [4,5]. This variability has increased interest in precision approaches that seek to identify characteristics associated with greater or lesser treatment response. However, apparent differences between subgroups should be interpreted cautiously, because post hoc subgroup patterns do not by themselves establish true treatment effect heterogeneity. Formal interaction analyses and prospective validation are required before a baseline characteristic can be considered predictive of response [6]. Baseline adiposity is therefore of particular interest as a candidate characteristic because it directly reflects the physiological burden targeted by body compositionbody composition interventions and may provide information that is not captured by BMI alone.

Food-derived bioactive peptides have attracted considerable interest as potential nutritional modulators of metabolic and body compositionbody composition-related processes. Experimental studies have reported effects on lipid metabolism, adipocyte differentiation, glucose handling, appetite-related signaling, and inflammatory pathways, although these activities depend strongly on peptide sequence, molecular size, source protein, processing conditions, and gastrointestinal stability [7,8]. Despite this expanding preclinical literature, translation into controlled human studies remains comparatively limited. In particular, evidence derived from isolated peptides or experimental hydrolysates cannot be assumed to apply directly to complex food-derived peptide preparations used in clinical settings. Human studies therefore require both adequate characterization of the investigational material and direct evaluation of clinically relevant outcomes [9].

Pumpkin seeds (*Cucurbita* spp.) contain proteins, lipids—including unsaturated fatty acids—dietary fiber, minerals, and phytochemicals. Their relative proportions depend on species, cultivar, and processing conditions. Seed proteins provide a starting material for peptide-containing preparations, but the composition of whole seeds cannot be used to infer the composition of a processed extract. Previous studies have characterized pumpkin seed proteins and experimentally prepared peptide fractions and identified candidate sequences with biological activities under defined laboratory conditions [10,11]. Sequence homology research has also described insulin receptor-binding peptide motifs in several plants, including pumpkin [12]. Together, these studies provide a molecular rationale for investigating pumpkin-seed-derived peptide preparations and support further clinical evaluation of standardized preparations, such as IRK-19^®^, for their potential effects on body composition.

The research team’s earlier animal investigations informed dose selection for the present trial, and functional screening of selected individual pumpkin-seed-derived peptides guided the choice of analytical markers. We evaluated the complete IRK-19^®^ preparation, rather than a purified peptide, in adults with overweight. Proximate composition, relative molecular weight distribution, and sequence-defined marker peptides were characterized alongside clinical assessment of body weight, BMI, and DXA-derived body composition. The primary comparison concerned the randomized study population; a separate, post hoc analysis explored body compositionbody composition responses according to baseline total fat mass. Metabolic and hematological results were assessed as supportive and safety-relevant outcomes, not as evidence of a human molecular mechanism.

## 2. Results

### 2.1. Investigational Material Characterization and Participant Disposition

**Figure 1:**
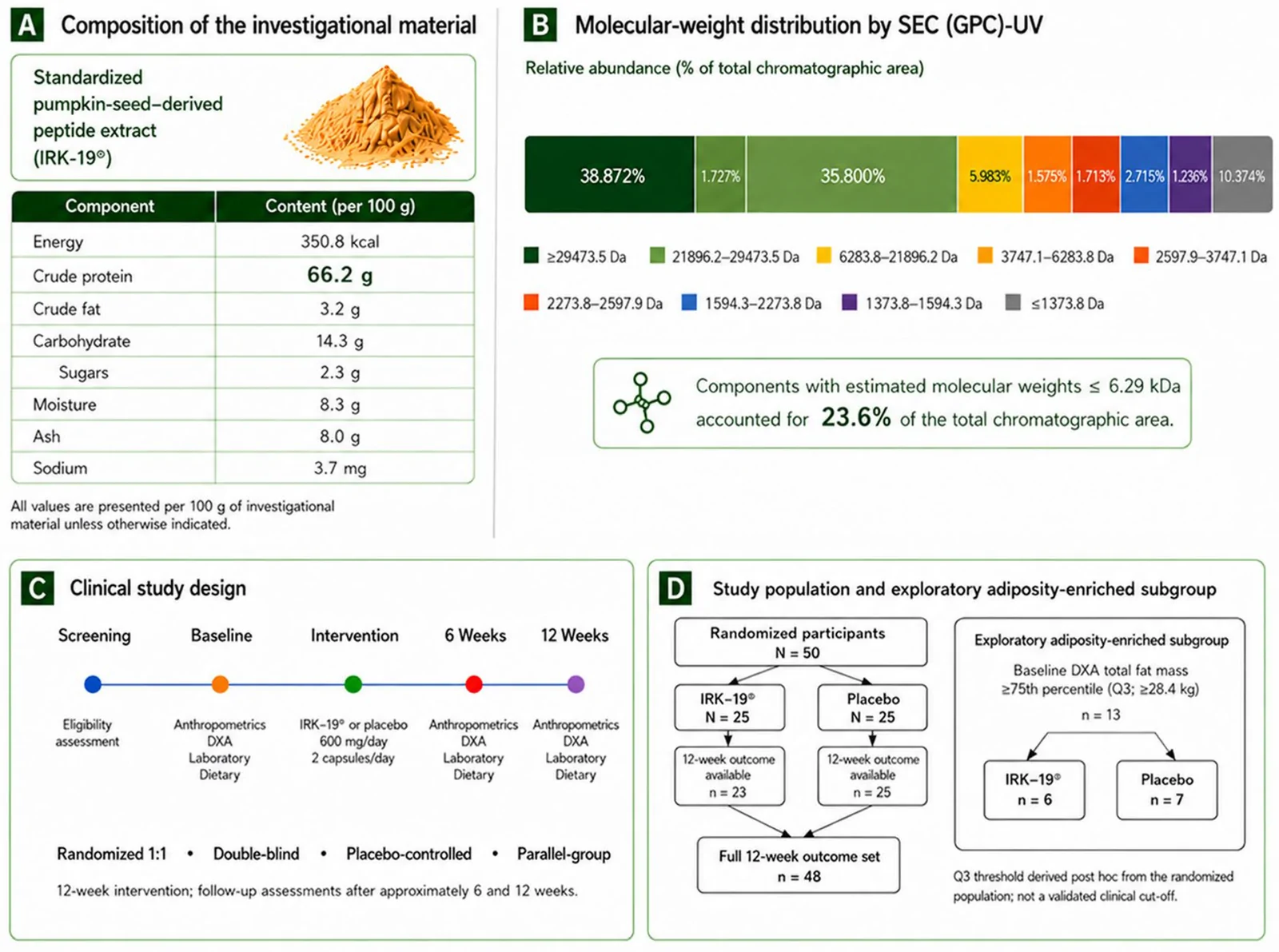
Characterization of the investigational material, clinical study design, and participant disposition. (**A**) Proximate composition of the administered pumpkin-seed-derived preparation. (**B**) Relative distribution of apparent molecular weights determined by SEC-UV. The apparent ≤6.29 kDa interval contributed 23.6% of total chromatographic area. (**C**) The 12-week randomized, double-blind, placebo-controlled trial included IRK-19^®^ and wheat-dextrin placebo (600 mg/day; two capsules/day in each group), with assessments at baseline and approximately 6 and 12 weeks. (**D**) Of 50 randomized participants (25 per group), 49 completed the intervention, and 48 had evaluable Week 12 DXA results (IRK-19^®^, *n* = 23; placebo, *n* = 25). The post hoc subgroup comprised 13 participants with baseline total fat mass ≥28.4 kg (IRK-19^®^, *n* = 6; placebo, *n* = 7). DXA, dual-energy X-ray absorptiometry; SEC-UV, size-exclusion chromatography with ultraviolet detection.

The investigational material was derived from pumpkin seeds and supplied as clinical production batch 2205100101. Proximate composition was 66.2 g/100 g crude protein, 3.2 g/100 g crude fat, 14.3 g/100 g carbohydrate, 8.3 g/100 g moisture, and 8.0 g/100 g ash (Figure 1A). Crude protein is a nitrogen-based compositional estimate, not a measurement of total peptide content. By size exclusion chromatography with ultraviolet detection (SEC-UV), components with apparent molecular weights ≤6.29 kDa accounted for 23.6% of the total chromatographic area (Figure 1B). This percentage is a relative UV-area index, not an absolute peptide mass fraction or measure of purity. Six candidate peptide markers were selected following the research team’s previous functional screening and identified by mass spectrometry. Their sequences were GGEQQDQEGEEERE, GGEQQDQEGEEEREAEREY, SRGGEQQDQEGEEE, SRGGEQQDQEGEEER, SRGGEQQDQEGEEEREAEREY, and NIIKVEGPL. Using HPLC with purified reference standards, the six selected markers had a combined measured content of 2.79% (w/w) in the clinical batch. This value is their sum, not the total peptide content of IRK-19^®^. The clinical study tested the complete preparation; these analytical data do not identify a single peptide responsible for clinical outcomes.

The study followed a 12-week randomized, double-blind, placebo-controlled, parallel-group design, with body compositionbody composition assessments performed at baseline and after 6 and 12 weeks of intervention (Figure 1C). Fifty participants were randomized in a 1:1 ratio to receive IRK-19^®^ or placebo (*n* = 25 per group). Forty-nine participants completed the 12-week intervention, and 48 participants had evaluable DXA data at the 12-week assessment (IRK-19^®^, *n* = 23; placebo, *n* = 25). For the post hoc exploratory adiposity-enriched analysis, the 75th percentile of baseline DXA total fat mass was 28.4 kg; 13 participants met this criterion, including 6 in the IRK-19^®^ group and 7 in the placebo group (Figure 1D). Baseline demographic, anthropometric, DXA-derived body compositionbody composition, and selected metabolic characteristics are summarized in Table 1. Participant screening and disposition are summarized in the CONSORT flow diagram (Supplementary Figure S5).

### 2.2. Overall Anthropometric and Body compositionBody Composition Responses

**Figure 2:**
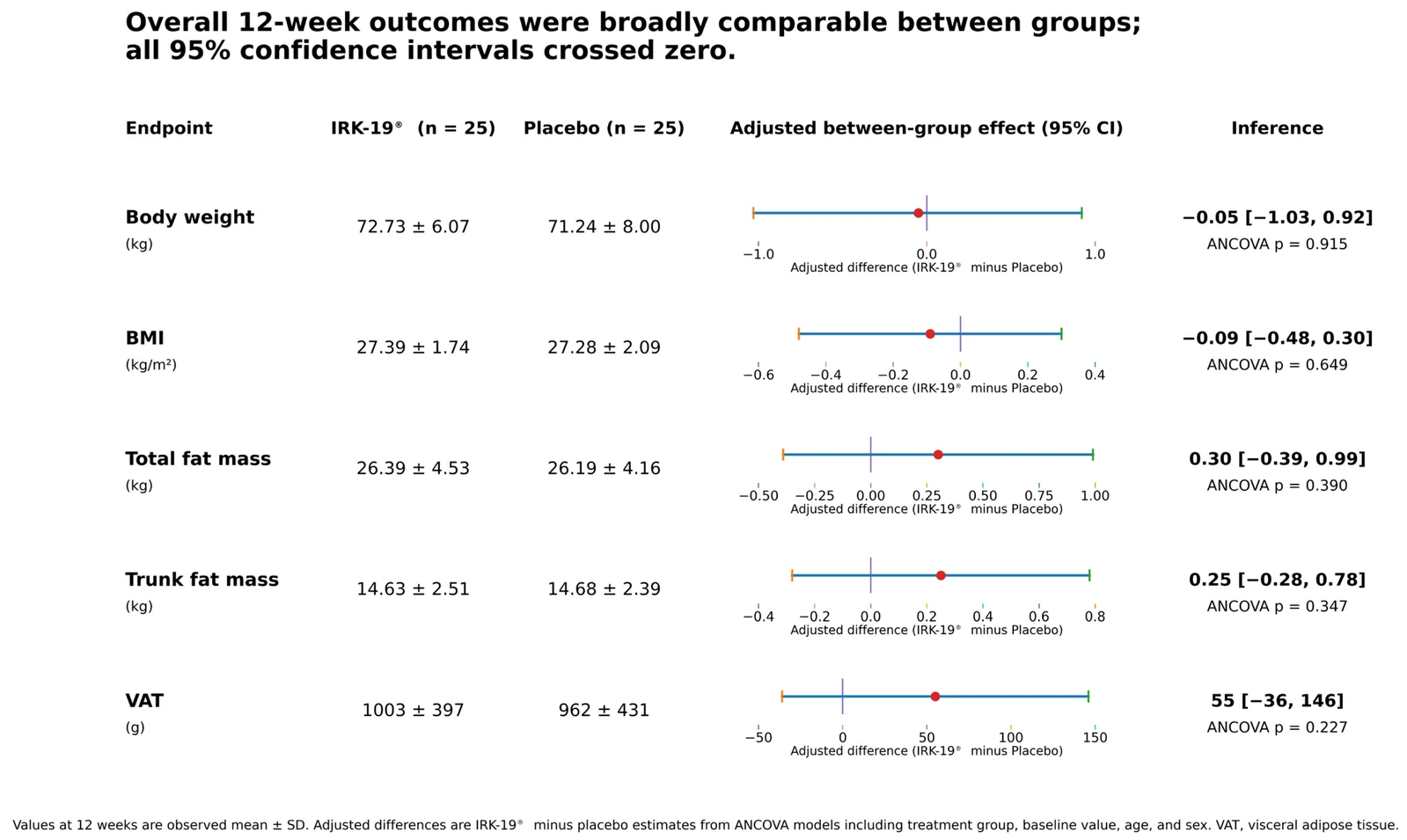
Overall anthropometric and body compositionbody composition responses after 12 weeks of intervention. Observed 12-week values for body weight, BMI, total fat mass, trunk fat mass, and visceral adipose tissue (VAT) mass are presented by treatment group. Adjusted between-group differences were estimated using ANCOVA with the corresponding baseline value, age, and sex as covariates and are expressed as IRK-19^®^ minus placebo with 95% confidence intervals.

In the overall analysis population, no clear between-group differences were observed in the selected anthropometric and DXA-derived body compositionbody composition outcomes after 12 weeks of intervention (Figure 2). The ANCOVA-adjusted between-group difference was −0.05 kg for body weight (95% CI, −1.03 to 0.92; *p* = 0.915) and −0.09 kg/m^2^ for BMI (95% CI, −0.48 to 0.30; *p* = 0.649). Adjusted differences were likewise small for total fat mass (0.30 kg; 95% CI, −0.39 to 0.99; *p* = 0.390), trunk fat mass (0.25 kg; 95% CI, −0.28 to 0.78; *p* = 0.347), and visceral adipose tissue mass (55 g; 95% CI, −36 to 146; *p* = 0.227).

No clear between-group differences were evident at the 6-week assessment. Adjusted between-group differences at this time point were 0.11 kg for body weight (95% CI, −0.50 to 0.73; *p* = 0.719), −0.01 kg/m^2^ for BMI (95% CI, −0.27 to 0.26; *p* = 0.962), and −0.04 kg for total fat mass (95% CI, −0.53 to 0.44; *p* = 0.857). Overall, the 6- and 12-week assessments did not demonstrate clear population-level between-group differences in the anthropometric or adiposity outcomes evaluated in the present analysis.

Dietary intake was assessed at Weeks 1 and 12 in the original clinical study. Mean daily energy intake in the IRK-19^®^ group was 1775.6 ± 204.0 kcal/day at Week 1 and 1783.4 ± 186.0 kcal/day at Week 12, compared with 1781.4 ± 180.7 and 1756.9 ± 179.4 kcal/day, respectively, in the placebo group. The between-group comparison of change in energy intake did not reach statistical significance (*p* = 0.183). Comparisons of changes in protein, fat, and carbohydrate intake likewise did not reach statistical significance (*p* = 0.879, 0.054, and 0.215, respectively). These group-level data neither establish dietary equivalence nor characterize dietary behavior specifically within the post hoc adiposity-enriched subgroup. Baseline activity was assessed using the IPAQ.

### 2.3. Metabolic and Hematological Laboratory Findings

Metabolic and hematological measurements were analyzed as supportive laboratory outcomes among participants with available data. At the 12-week blood test assessment, 49 participants had evaluable samples (IRK-19^®^, *n* = 24; placebo, *n* = 25); this laboratory analysis population differed from the 48 participants with evaluable 12-week DXA measurements. The adjusted between-group difference in fasting glucose (IRK-19^®^ minus placebo) was +9.00 mg/dL (95% CI, 1.93 to 16.07; *p* = 0.014). Observed group means at baseline and Week 12 were 98.24 and 98.88 mg/dL in the IRK-19^®^ group and 97.56 and 90.32 mg/dL in the placebo group, respectively. The adjusted between-group difference reflects the comparison between treatment groups after covariate adjustment rather than a within-group change in the IRK-19^®^ group. At Week 12, fasting glucose ≥126 mg/dL was recorded in 2/24 IRK-19^®^ participants and 0/25 placebo participants. No fasting glucose value <70 mg/dL was observed in either group at the available assessments. The corresponding HOMA-IR-adjusted difference at Week 12 was 0.44 (95% CI, −0.08 to 0.96; *p* = 0.093).

For triglycerides, observed means at baseline and Week 12 were 138.00 and 186.67 mg/dL in the IRK-19^®^ group and 116.28 and 108.92 mg/dL in the placebo group, respectively. The adjusted between-group difference at Week 12 was +70.16 mg/dL (95% CI, 9.90 to 130.41; *p* = 0.024). At Week 12, triglycerides ≥500 mg/dL were recorded in 2/24 IRK-19^®^ participants and 0/25 placebo participants. Total cholesterol, HDL-C, and LDL-C did not show statistically significant adjusted between-group differences at Week 12. Observed mean platelet counts at baseline and Week 12 were 290.24 and 281.25 × 10^3^/µL in the IRK-19^®^ group and 282.72 and 290.20 × 10^3^/µL in the placebo group, respectively. The adjusted between-group difference was −17.62 × 10^3^/µL (95% CI, −33.81 to −1.43; *p* = 0.034). No participant in either group had a Week 12 platelet count <150 × 10^3^/µL. White blood cell count, red blood cell count, hemoglobin, hematocrit, and other erythrocyte indices did not show statistically significant differences between groups at Week 12. These nominal laboratory findings were obtained across multiple tested outcomes without multiplicity adjustment.

### 2.4. Body Composition Responses Associated with Higher Baseline Adiposity

**Figure 3:**
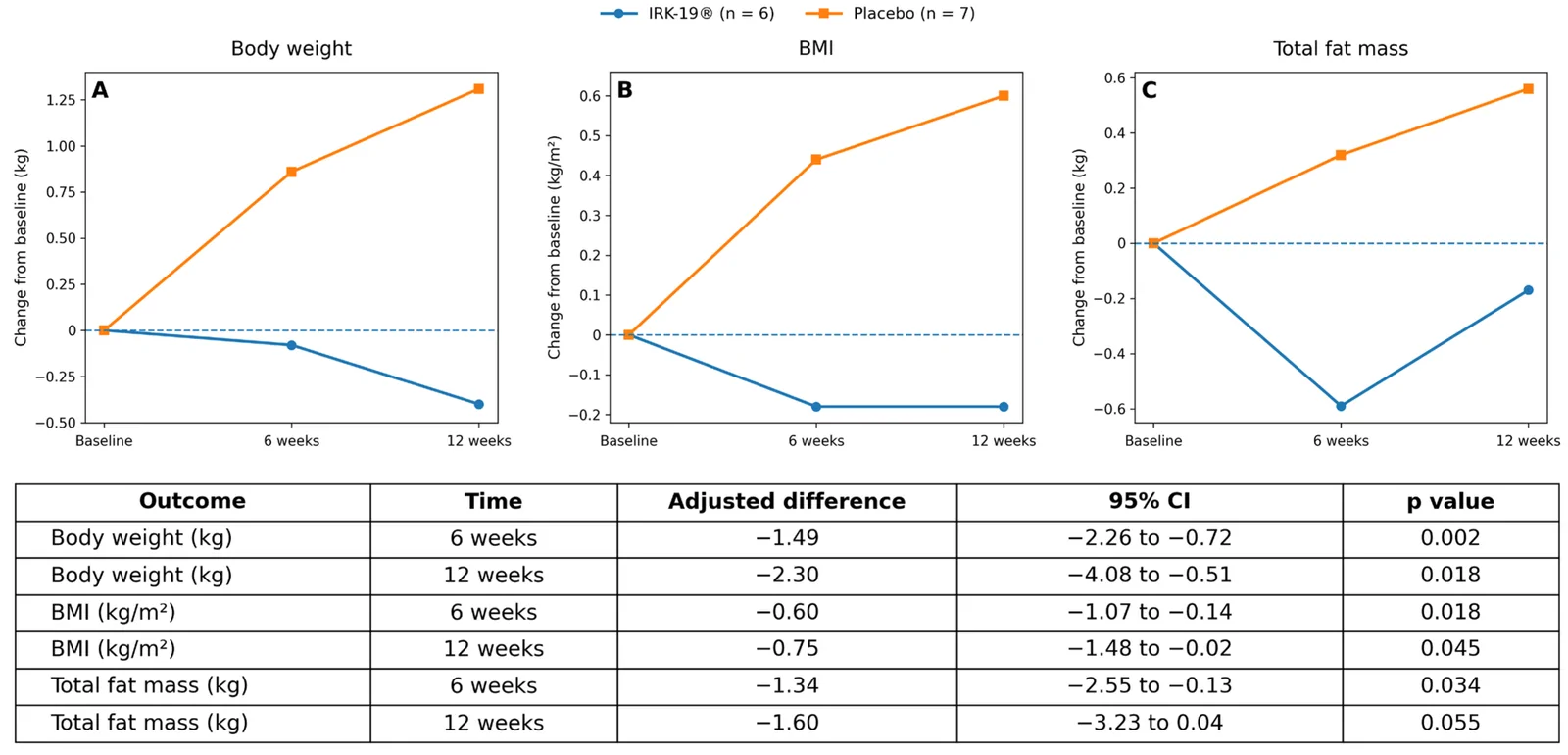
Body compositionBody composition responses in the exploratory adiposity-enriched subgroup. Participants with baseline DXA total fat mass at or above the study-specific 75th percentile (Q3; ≥28.4 kg) were included in this post hoc exploratory analysis (IRK-19^®^, *n* = 6; placebo, *n* = 7). Panels (**A**–**C**) display the observed, unadjusted group-mean changes from baseline in body weight, BMI, and total fat mass at 6 and 12 weeks. The accompanying between-group estimates are ANCOVA-adjusted differences (IRK-19^®^ minus placebo), with 95% confidence intervals, adjusted for the corresponding baseline outcome, age, and sex. Plotted observed changes and adjusted between-group estimates are different statistical quantities and must not be interpreted as interchangeable. The 28.4 kg threshold was derived post hoc from this sample and is not a validated clinical cut-off. DXA, dual-energy X-ray absorptiometry; Q3, third quartile; BMI, body mass index; CI, confidence interval. The reported *p* values are nominal and were not adjusted for multiplicity.

In the post hoc exploratory adiposity-enriched subgroup, defined by baseline DXA total fat mass at or above the 75th percentile (≥28.4 kg; *n* = 13), a more pronounced pattern of between-group differences was observed than in the overall population (Figure 3). After six weeks, the adjusted between-group difference in body weight was −1.49 kg (95% CI, −2.26 to −0.72; *p* = 0.002). The between-group difference in body weight remained evident at Week 12, with an adjusted estimate of −2.30 kg (95% CI, −4.08 to −0.51; *p* = 0.018). BMI showed a similar pattern, with adjusted differences of −0.60 kg/m^2^ after 6 weeks (95% CI, −1.07 to −0.14; *p* = 0.018) and −0.75 kg/m^2^ after 12 weeks (95% CI, −1.48 to −0.02; *p* = 0.045).

DXA-derived total fat mass showed a similar pattern. After six weeks, total fat mass decreased by 0.59 kg from baseline in the IRK-19^®^ group and increased by 0.32 kg in the placebo group, corresponding to an adjusted between-group difference of −1.34 kg (95% CI, −2.55 to −0.13; *p* = 0.034). After 12 weeks, the observed changes were −0.17 kg and +0.56 kg in the IRK-19^®^ and placebo groups, respectively, with an adjusted between-group difference of −1.60 kg (95% CI, −3.23 to 0.04; *p* = 0.055) (Figure 3; Table 2). In complementary analyses treating baseline total fat mass as a continuous variable, the treatment-by-baseline-adiposity interaction estimates were directionally consistent with larger estimated between-group differences at higher baseline fat mass, although the interactions did not reach statistical significance for body weight (*p* = 0.065), BMI (*p* = 0.106), or total fat mass (*p* = 0.057). Accordingly, the adiposity-enriched findings should be regarded as hypothesis-generating and as support for prospective evaluation of baseline adiposity as a potential enrichment characteristic rather than as evidence of a validated treatment response threshold.

### 2.5. Safety and Adverse Events

According to the clinical study completion report and adverse event records, no adverse events (AEs) or serious adverse events (SAEs) were recorded in either randomized group during the intervention period, and no discontinuation was attributed to a recorded AE. These clinical event records should be distinguished from the laboratory abnormalities described in Section 2.3. The available dataset did not contain confirmatory testing or documented investigator assessments for individual elevated glucose and triglyceride results. Accordingly, an absence of recorded AEs cannot be taken to establish the absence of laboratory safety concerns or to exclude a possible relationship to the intervention. Systematic longitudinal liver and renal function results were not available in the trial records examined for this reanalysis, and no conclusions regarding hepatic or renal safety are drawn from this dataset.

## 3. Discussion

### 3.1. Principal Findings and Working Hypothesis

This randomized controlled trial yielded two complementary observations regarding body compositionbody composition outcomes. In the overall study population, between-group comparisons of body weight, BMI, and DXA-derived adiposity measures did not reach statistical significance. Further exploratory analyses by baseline total fat mass, however, revealed a different pattern of response. Among participants with higher baseline total fat mass, adjusted between-group differences at Week 6 favored IRK-19^®^ for body weight, BMI, and total fat mass. At Week 12, the between-group differences in body weight and BMI remained nominally statistically significant. The estimated difference in total fat mass was in the same direction, although its 95% confidence interval included zero.

These observations suggest that baseline fat mass may be a physiological characteristic worth considering in future investigations of variation in body compositionbody composition responses. However, the subgroup analysis was conducted post hoc and was not adjusted for multiple comparisons, and formal treatment-by-baseline-fat-mass interaction tests did not reach statistical significance. The findings should therefore be regarded as exploratory and do not establish baseline fat mass as a predictor of treatment response. Future trials with prespecified adiposity strata and adequate sample sizes could further evaluate body compositionbody composition responses to IRK-19^®^ across different levels of baseline fat mass.

### 3.2. Baseline Adiposity and Heterogeneity in Intervention Response

Heterogeneity in response to nutritional and weight management interventions is increasingly recognized as an important feature of obesity research. Individuals with similar BMI values may differ substantially in absolute fat mass, regional adiposity, adipose tissue function, and metabolic phenotype, all of which may influence the magnitude of response to a given intervention [3,4,5]. Importantly, substantial inter-individual variability has also been observed in controlled human weight management studies. In the DIETFITS randomized clinical trial, participants assigned to the same dietary intervention exhibited a wide range of individual weight changes despite relatively modest differences in mean responses between dietary strategies [13]. A phenotype-tailored lifestyle intervention in adults with obesity subsequently showed greater weight loss than a standard lifestyle program in a proof-of-concept study [14], while a randomized trial of personalized nutrition demonstrated that incorporating individual metabolic responses and other personal characteristics could improve selected cardiometabolic outcomes compared with generalized dietary advice [15]. Together, these studies support the broader concept that population average responses may obscure meaningful inter-individual differences and that baseline phenotypic characteristics warrant consideration when evaluating intervention efficacy.

Further exploratory stratification using DXA-measured baseline total fat mass revealed a pattern of body compositionbody composition responses in participants with higher adiposity that differed from the overall study population. This observation is conceptually consistent with recent obesity frameworks emphasizing the value of directly assessing adiposity to capture physiological information that BMI alone may not adequately reflect [1,2,3]. The 28.4 kg threshold was derived post hoc from the study-specific 75th percentile of baseline total fat mass, and formal treatment-by-baseline-fat-mass interaction tests did not reach statistical significance. Baseline fat mass therefore remains a candidate characteristic for further investigation rather than an established predictor of treatment response. Nevertheless, the similar directions of the subgroup estimates and continuous variable analyses provide a rationale for prospective trials with prespecified adiposity strata to examine the relationship between baseline fat mass and body compositionbody composition responses to IRK-19^®^.

### 3.3. Food-Derived Peptides: Biological Plausibility and Translational Evidence

Food-derived bioactive peptides have been investigated for their potential roles in metabolic regulation and body composition. Experimental studies have reported effects on lipid metabolism, adipocyte differentiation, glucose homeostasis, appetite-related signaling, and inflammatory pathways [7,8]. The activity of these peptides may depend on their sequences, molecular size, gastrointestinal stability, intestinal absorption, and the composition of the administered preparation [9,16]. These considerations are relevant to IRK-19^®^, a standardized pumpkin-seed-derived preparation containing multiple peptide species rather than a single purified peptide.

Previous investigations of pumpkin-seed proteins, hydrolysates, and candidate peptides provide a basis for studying pumpkin-derived peptide preparations [10,11]. Related putative insulin receptor-binding peptide motifs have also been described in pumpkin and other plants [12]. In the development of IRK-19^®^, the research team conducted functional screening of selected individual peptides and used the results to prioritize six sequence-defined markers for analytical characterization. The detailed functional findings are being reported separately and are not included in the present clinical study. Although these investigations informed the selection of candidate peptides, the contributions of individual constituents to the activity of the complete preparation have not been established. Defined combinations of the selected peptides have not been evaluated in dedicated mixture experiments. Moreover, the present clinical trial assessed the complete IRK-19^®^ preparation rather than individually administered purified peptides. Further studies of gastrointestinal stability, systemic exposure, and component interactions are therefore needed to determine how specific peptide constituents may contribute to the observed clinical responses.

Clinical studies of other food-derived peptide preparations provide additional context for interpreting body composition outcomes. In a randomized, double-blind, placebo-controlled trial involving adults with overweight, a peptide-rich whey protein hydrolysate was associated with reductions in waist circumference at selected follow-up assessments, whereas the prespecified BMI outcome did not show a statistically significant between-group difference [17]. Although that preparation differs from IRK-19^®^ in source and composition, the findings illustrate the value of assessing several complementary anthropometric and body composition outcomes rather than relying on BMI alone.

Recent human evidence further illustrates that responses to food-derived peptide preparations may not be expressed uniformly across conventional obesity-related endpoints. In a randomized, double-blind, placebo-controlled trial of adults with overweight, a peptide-rich whey protein hydrolysate showed endpoint-specific responses, with reductions in waist circumference at selected follow-up assessments despite no significant difference in the prespecified BMI outcome [17]. Although that intervention differs substantially from IRK-19^®^ in source, composition, and peptide profile, the study illustrates a relevant translational principle: peptide-based nutritional interventions may produce more discernible responses in selected anthropometric or body composition domains than across all conventional weight-related outcomes.

### 3.4. Translational Implications and Population Enrichment

From a translational perspective, the present findings suggest that future evaluation of pumpkin-seed-derived peptide interventions may benefit from participant selection strategies that incorporate direct measures of adiposity rather than relying exclusively on BMI. Contemporary precision-nutrition and obesity frameworks increasingly recognize that individuals meeting the same BMI-based classification may differ substantially in physiological phenotype and intervention responsiveness [1,4,5,13,14,15]. In this context, baseline total fat mass may represent a useful candidate enrichment variable when the intended outcome is modification of body composition. Enriching future study populations with participants with greater baseline adiposity could potentially reduce physiological heterogeneity and improve the precision with which treatment-associated changes are estimated in prospective trials.

Importantly, the present findings do not establish the study-derived threshold of 28.4 kg total fat mass as a clinically actionable cut-off. Rather, this threshold provides an empirical basis for designing a prospective validation study in which adiposity strata and treatment-by-adiposity interactions are predefined. Such studies should determine whether absolute total fat mass, percentage body fat, regional fat distribution, visceral adiposity, or combinations of these measures provide the most reproducible means of identifying populations in whom a pumpkin-seed-derived peptide intervention may show a more discernible body composition response. This approach would shift the development strategy from evaluating population-average responses in broadly defined adults with overweight toward prospectively testing whether defined phenotypic characteristics can improve population selection and the interpretation of intervention responses.

### 3.5. Interpretation of Metabolic and Hematological Laboratory Findings

Metabolic and hematological laboratory findings provide additional context for evaluating the clinical tolerability of IRK-19^®^. At Week 12, the adjusted fasting glucose estimate was higher in the IRK-19^®^ group than in the placebo group. The observed mean glucose level changed only modestly from baseline in the IRK-19^®^ group, whereas the placebo-group mean decreased. Individual glucose measurements nevertheless warrant consideration alongside these group-level results. The between-group difference in triglycerides was accompanied by Week 12 values ≥500 mg/dL in two participants receiving IRK-19^®^. No corresponding statistically significant between-group differences were identified in fasting insulin, HOMA-IR, or the other lipid variables. These findings should be interpreted together, while recognizing that the absence of a consistent pattern across metabolic markers does not resolve the clinical significance or potential relationship to the intervention of individual glucose and triglyceride elevations.

The adjusted between-group difference in platelet count at Week 12 was not accompanied by a platelet count below 150 × 10^3^/µL in either group. No adverse events or serious adverse events were recorded in the study’s event records. Nevertheless, clinical event reporting and laboratory assessments provide complementary information and should be considered together when evaluating tolerability. The present findings do not establish whether individual biochemical changes were related to the study intervention. Given the number of laboratory outcomes assessed and the relatively small sample size, future trials should include prespecified monitoring of metabolic and hematological parameters, together with appropriate hepatic and renal assessments, to further characterize the clinical safety profile of IRK-19^®^.

### 3.6. Strengths, Limitations, and Future Directions

The randomized, double-blind, placebo-controlled design and repeated DXA assessments provided a framework for evaluating body compositionbody composition outcomes beyond body weight and BMI. The investigational preparation was characterized using compositional analysis, SEC-UV profiling, and selected peptide markers, linking the clinical evaluation to a defined production batch. The study also examined body compositionbody composition responses at both the overall population and exploratory subgroup levels. In participants with higher baseline total fat mass, the subgroup estimates showed a consistent direction across several outcomes and assessment time points. Continuous treatment-by-baseline-fat-mass analyses were conducted to examine this observation without relying solely on the study-derived subgroup threshold.

The exploratory subgroup comprised 13 participants and was defined post hoc using the study-specific 75th percentile of baseline total fat mass (28.4 kg). Its estimates should therefore be interpreted in the context of the small subgroup size, the absence of multiplicity adjustment, and the nonsignificant formal treatment-by-baseline-fat-mass interactions. The available protocol materials did not document a formal a priori sample-size calculation, and the trial was not prospectively powered to assess treatment effect heterogeneity by baseline adiposity. Accordingly, the subgroup findings identify a question for prospective investigation rather than establishing baseline fat mass as a predictor of response. Similarly, the overall population results should not be interpreted as definitive evidence that clinically meaningful differences are absent.

The 12-week study period allowed assessment of body compositionbody composition responses at Weeks 6 and 12. Longer follow-up is needed to determine the persistence of the observed response patterns and to characterize longer-term tolerability. Both groups received structured dietary counseling, and group-level comparisons of dietary changes did not reach statistical significance. Nevertheless, the available data do not establish dietary equivalence within the small exploratory subgroup. Quantitative adherence summaries and verified longitudinal physical-activity data were also unavailable for the present reanalysis. Future trials would benefit from prespecified adiposity strata, an appropriate sample-size calculation, and more detailed monitoring of supplement adherence, dietary intake, and physical activity.

The present trial evaluated the complete IRK-19^®^ preparation rather than isolated peptide constituents. Previous functional screening informed the selection of candidate peptide markers, but peptide-specific gastrointestinal stability, systemic exposure, and molecular mechanisms were not directly assessed in this clinical study. Further investigation of individual peptides and defined combinations in appropriate experimental models, together with exposure and mechanistic assessments, could help clarify the biological processes underlying responses to the complete preparation. Future clinical studies should also incorporate prespecified metabolic, hematological, hepatic, and renal monitoring to further characterize its safety profile.

## 4. Materials and Methods

### 4.1. Investigational Material and Physicochemical Characterization

IRK-19^®^ was supplied by Greenyn Biotechnology Co., Ltd. (Taichung, Taiwan) as a standardized pumpkin-seed-derived preparation produced by a process incorporating supercritical CO_2_ extraction. The clinical trial used production batch 2205100101. Proximate analysis measured crude protein, crude fat, carbohydrate, moisture, and ash. Six candidate peptide markers were selected following earlier functional screening of individual peptides and identified by mass spectrometry. Their combined content was measured by HPLC using purified reference standards; individual marker concentrations and total peptide content were not established for this trial. The material was a complex preparation rather than a purified peptide.

Molecular weight distribution was characterized by SEC-UV using a Waters Alliance e2695 Separations Module equipped with a Waters 2998 Photodiode Array (PDA) Detector (Waters Corporation, Milford, MA, USA), with UV detection at 220 nm. A PSS PROTEEMA guard column (5 μm, 8 × 50 mm) coupled to a PSS PROTEEMA column (5 μm, 100 Å, 8 × 300 mm) (PSS Polymer Standards Service GmbH, Mainz, Germany). Analysis was performed using a phosphate–NaCl mobile phase at pH 6.6, a flow rate of 1.0 mL/min, a column temperature of 25 °C, and UV detection at 220 nm. Molecular weight calibration was established using angiotensin II (1.0 kDa), neurotensin (1.7 kDa), aprotinin (6.7 kDa), myoglobin (17 kDa), and ovalbumin (45 kDa), based on the relationship between retention time and log-transformed molecular weight. Relative chromatographic areas within predefined molecular weight intervals were used to describe the molecular weight distribution of the preparation and were not interpreted as absolute peptide concentrations.

### 4.2. Study Design, Participants, and Intervention

This was a 12-week, single-center, randomized, double-blind, placebo-controlled, parallel-group clinical trial conducted at Wan-Fang Hospital, Taipei, Taiwan. Participants were recruited between 14 November 2022 and 27 December 2023. The trial was registered at ClinicalTrials.gov (NCT05542160; first submitted on 13 September 2022 and first posted on 15 September 2022) and conducted under study protocol G-CAZ0040161110628-1. The protocol was reviewed and approved by the TMU-Joint Institutional Review Board (approval No. N202205040; approval date: 3 August 2022). The study was conducted in accordance with the Declaration of Helsinki [18], and written informed consent was obtained from all participants before participation in any study-related procedures. The present study is reported in accordance with the CONSORT 2025 statement [19].

Adults aged 20–60 years were eligible if they had a body mass index (BMI) of 25–30 kg/m^2^ and a waist circumference >80 cm. Major exclusion criteria included pregnancy or lactation, recent childbirth, use of medications considered likely to affect body weight, recent bariatric or major surgery, body weight change >5% during the preceding 3 months, severe cardiovascular disorders, active cancer treatment or end-of-life status, and recent participation in another weight control clinical trial. No glycemic criterion was used for participant enrollment.

Eligible participants were randomly assigned in a 1:1 ratio to IRK-19^®^ or placebo. A central coordinator responsible for allocation concealment performed the computer-generated randomization using blocks of four with stratification by sex. The randomization records were retained by a designated research assistant. A separate research assistant handled participant enrollment and dispensed the assigned intervention according to the allocation procedure without advance access to group assignments. Participants and outcome assessors were blinded to treatment assignment. The daily IRK-19^®^ dose of 600 mg was selected by converting the effective dose observed in the research team’s previous animal study to a human equivalent dose. Participants received either IRK-19^®^ (600 mg/day) or wheat dextrin placebo (600 mg/day), administered as two capsules (300 mg/capsule) per day for 12 weeks. The active and placebo preparations were encapsulated in identical white, opaque, plant-based capsule shells and sealed to prevent visual identification of the contents; equivalence in capsule odor was also confirmed.

### 4.3. Dietary Management, Physical Activity, and Supplement Adherence

Both treatment groups received the same structured dietary education and counseling from a research dietitian, with guidance aimed at approximately 3–5% body weight reduction during the intervention. Dietary questionnaires were completed before and during follow-up, and the available nutritional summaries compared daily energy and macronutrient intake at Weeks 1 and 12. These dietary data were analyzed at the group level and were not considered evidence of dietary equivalence within the exploratory subgroup. Baseline physical activity was assessed using the International Physical Activity Questionnaire (IPAQ); longitudinal IPAQ comparisons could not be verified from the records available for the present reanalysis.

Supplement intake was monitored using daily pill intake logs and regular contact by the study team.

### 4.4. Anthropometric, Body compositionBody Composition, and Laboratory Assessments

The original study protocol defined evaluation of weight reduction as the primary objective, with reduction in body fat and improvement in blood lipids as secondary objectives. The registered study specified anthropometric, body compositionbody composition, and biochemical assessments at baseline and after 6 and 12 weeks. The present reanalysis focuses on body weight, BMI, DXA-derived body compositionbody composition measures, and selected laboratory variables available at the individual participant level.

Clinical assessments were performed at baseline and at two post-baseline assessments corresponding to approximately 6 and 12 weeks of intervention. These visits were operationally designated as Weeks 7 and 13 in the original study records and are referred to as the 6-week and 12-week assessments throughout the present manuscript to reflect elapsed intervention time.

Whole-body composition was assessed by dual-energy X-ray absorptiometry (DXA) using a Lunar Prodigy system (version 9.1; GE Healthcare, Waukesha, WI, USA) at Wan-Fang Hospital. All examinations were performed using the same scanner by an experienced radiological technologist who was blinded to treatment allocation. DXA-derived measures available in the study dataset included total fat mass, trunk fat mass, total lean mass, and visceral adipose tissue (VAT) mass. The present outcome analyses focused on total fat mass, trunk fat mass, and VAT mass. Body weight and BMI were assessed at the corresponding study visits. The available records did not permit verification of a study-specific phantom calibration schedule or an instrument-specific coefficient of variation.

Fasting laboratory measures available for the present reanalysis included glucose, insulin, triglycerides, total cholesterol, HDL-C, LDL-C, and complete blood-cell-count parameters. Homeostasis Model Assessment of Insulin Resistance (HOMA-IR) was calculated as fasting glucose (mg/dL) × fasting insulin (µIU/mL)/405 [20]. For descriptive safety assessment, individual Week 12 values were compared with reporting thresholds of fasting glucose ≥126 mg/dL, triglycerides ≥500 mg/dL, and platelet count <150 × 10^3^/µL. These single measurements were not used to assign clinical diagnoses or establish causality. Fasting glucose values <70 mg/dL and <54 mg/dL were also examined to describe biochemical hypoglycemia [21].

Adverse events were recorded during the intervention in the study case report forms and adverse event records. The absence of recorded clinical adverse events was not assumed to indicate the absence of laboratory abnormalities; clinical events and laboratory findings were therefore reported separately.

### 4.5. Statistical Analysis

Continuous variables were presented as mean ± standard deviation (SD), and categorical variables as counts and percentages. Baseline characteristics were summarized descriptively by randomized treatment group.

Between-group differences in body weight, body mass index (BMI), DXA-derived body compositionbody composition measures, and selected laboratory outcomes were evaluated at Weeks 6 and 12 using analysis of covariance (ANCOVA). Each model included the corresponding follow-up outcome as the dependent variable, treatment group as the main factor, and the baseline value of the outcome, age, and sex as covariates. Adjusted between-group differences were reported as IRK-19^®^ minus placebo, with two-sided 95% confidence intervals (CIs). Participants were analyzed according to their randomized group when both baseline and corresponding follow-up measurements were available. Missing values were not imputed.

The principal body compositionbody composition analyses assessed body weight, BMI, total fat mass, trunk fat mass, and visceral adipose tissue (VAT) mass in the overall study population. Laboratory outcomes were evaluated as supportive analyses. To explore whether body compositionbody composition responses varied with baseline adiposity, a post hoc subgroup was defined using the 75th percentile of baseline DXA-derived total fat mass in the randomized population. The resulting threshold of 28.4 kg identified 13 participants (IRK-19^®^, *n* = 6; placebo, *n* = 7). Between-group differences within this subgroup were estimated using the same ANCOVA approach as in the overall analysis.

Baseline total fat mass was also examined as a continuous variable in treatment-by-baseline-fat-mass interaction models for Week 12 body weight, BMI, and total fat mass. The body weight and BMI models included treatment group, the corresponding baseline outcome, baseline total fat mass, the treatment × baseline total fat mass interaction, age, and sex. The total-fat-mass model included treatment group, baseline total fat mass, the treatment × baseline total fat mass interaction, age, and sex. Interaction estimates were expressed per 5 kg increase in baseline total fat mass.

All statistical tests were two-sided, with *p* < 0.05 used as the nominal threshold for statistical significance. No adjustment for multiple comparisons was applied; therefore, findings from the post hoc subgroup, interaction, and laboratory analyses were interpreted as exploratory. Statistical analyses were performed using Python version 3.13 and statsmodels version 0.14.6.

## 5. Conclusions

In this randomized, double-blind, placebo-controlled trial, exploratory analyses identified nominally statistically significant between-group differences in body weight and BMI at Week 12 among participants with higher baseline total fat mass. These observations provide a basis for further investigation of body compositionbody composition responses to IRK-19^®^ in relation to baseline adiposity. The response pattern observed in this subgroup warrants further investigation, while between-group comparisons in the overall study population did not reach statistical significance. Because the subgroup was defined post hoc and formal treatment-by-baseline-fat-mass interaction tests were not statistically significant, the subgroup findings remain exploratory. Prospective trials with prespecified adiposity strata are needed to determine whether this response pattern can be reproduced and whether baseline fat mass has predictive value for treatment response. Individual changes in fasting glucose and triglyceride measurements also warrant further evaluation to clarify their clinical significance and potential relationship to the intervention.

Future adequately powered trials incorporating prespecified adiposity strata, comprehensive characterization of the investigational material, quantified supplement adherence, and appropriate metabolic and organ safety monitoring will help further define the clinical response to IRK-19^®^ across different levels of baseline adiposity.

## Figures and Tables

**Table 1:** Baseline characteristics of the randomized population.

Variable	IRK-19^®^ (*n* = 25)	Placebo (*n* = 25)
Age (years)	43.36 ± 8.89	45.00 ± 7.03
Male, *n* (%)	7 (28.0)	6 (24.0)
Body weight (kg)	72.53 ± 5.66	71.20 ± 7.78
BMI (kg/m^2^)	27.50 ± 1.68	27.23 ± 1.87
Total fat mass (kg)	26.31 ± 4.17	26.46 ± 3.38
Trunk fat mass (kg)	14.53 ± 2.27	14.87 ± 2.10
VAT mass (g)	963 ± 397	992 ± 460
Total lean mass (kg)	43.82 ± 5.96	42.30 ± 6.38
Fasting glucose (mg/dL)	98.24 ± 13.76	97.56 ± 20.09
Insulin (µIU/mL)	9.93 ± 3.57	8.63 ± 3.08
HOMA-IR	2.46 ± 1.19	2.11 ± 0.97
Triglycerides (mg/dL)	138.00 ± 69.70	116.28 ± 60.92
HDL-C (mg/dL)	50.44 ± 9.73	58.00 ± 21.98
LDL-C (mg/dL)	120.08 ± 34.07	115.56 ± 34.61

Values are presented as mean ± SD. BMI, body mass index; VAT, visceral adipose tissue; HOMA-IR, homeostasis model assessment of insulin resistance; HDL-C, high-density lipoprotein cholesterol; LDL-C, low-density lipoprotein cholesterol. HOMA-IR was derived from fasting glucose and insulin concentrations.

**Table 2:** Exploratory comparison of adjusted between-group differences in the overall and adiposity-enriched populations.

Outcome	Analysis Population	6-Week Adjusted Difference (95% CI)	*p* Value	12-Week Adjusted Difference (95% CI)	*p* Value
Body weight (kg)	Overall population	0.11 (−0.50 to 0.73)	0.719	−0.05 (−1.03 to 0.92)	0.915
	Adiposity-enriched subgroup	−1.49 (−2.26 to −0.72)	0.002	−2.30 (−4.08 to −0.51)	0.018
BMI (kg/m^2^)	Overall population	−0.01 (−0.27 to 0.26)	0.962	−0.09 (−0.48 to 0.30)	0.649
	Adiposity-enriched subgroup	−0.60 (−1.07 to −0.14)	0.018	−0.75 (−1.48 to −0.02)	0.045
Total fat mass (kg)	Overall population	−0.04 (−0.53 to 0.44)	0.857	0.30 (−0.39 to 0.99)	0.390
	Adiposity-enriched subgroup	−1.34 (−2.55 to −0.13)	0.034	−1.60 (−3.23 to 0.04)	0.055

Table note: Adjusted between-group differences are expressed as IRK-19^®^ minus placebo and were estimated using ANCOVA models including treatment group, the corresponding baseline value, age, and sex. Negative estimates indicate lower values in the IRK-19^®^ group relative to placebo. The overall analysis included participants with available data at the corresponding assessment; 48 participants contributed to the 12-week body composition analysis (IRK-19^®^, *n* = 23; placebo, *n* = 25). The adiposity-enriched subgroup was defined post hoc as participants with baseline DXA total fat mass ≥75th percentile (Q3; ≥28.4 kg; *n* = 13; IRK-19^®^, *n* = 6; placebo, *n* = 7). Subgroup analyses were exploratory, and *p* values were not adjusted for multiplicity. BMI, body mass index; CI, confidence interval; DXA, dual-energy X-ray absorptiometry; Q3, third quartile.

## Data Availability

The de-identified individual participant data underlying this study were provided to the research team under a confidentiality agreement. The research team is not authorized to redistribute these data to third parties. Accordingly, individual participant data are not available to external researchers upon request.

## Supplementary Materials

The following supporting information can be downloaded at: https://www.mdpi.com/article/10.3390/ijms27198813/s1.

## Abbreviations

The following abbreviations are used in this manuscript:

ANCOVA	Analysis of covariance
BMI	Body mass index
CI	Confidence interval
CONSORT	Consolidated Standards of Reporting Trials
DXA	Dual-energy X-ray absorptiometry
HDL-C	High-density lipoprotein cholesterol
HOMA-IR	Homeostasis model assessment of insulin resistance
LDL-C	Low-density lipoprotein cholesterol
Q3	Third quartile (75th percentile)
SD	Standard deviation
SEC-UV	Size-exclusion chromatography with ultraviolet detection
VAT	Visceral adipose tissue
