# Supplementary material for: A Randomized Clinical Trial of a Standardized Pumpkin-Seed-Derived Peptide Extract: Body Composition Outcomes and Exploratory Analysis by Baseline Adiposity in Overweight Adults
Source: Int J Mol Sci. 2026 Oct 1;27(19):8813. doi: 10.3390/ijms27198813 (PMC13665551; doi:10.3390/ijms27198813)
Supplement: Supplementary file 1 [file ijms-27-08813-s001.zip › ijms-4546194-supplementary.pdf]

Supplementary Data

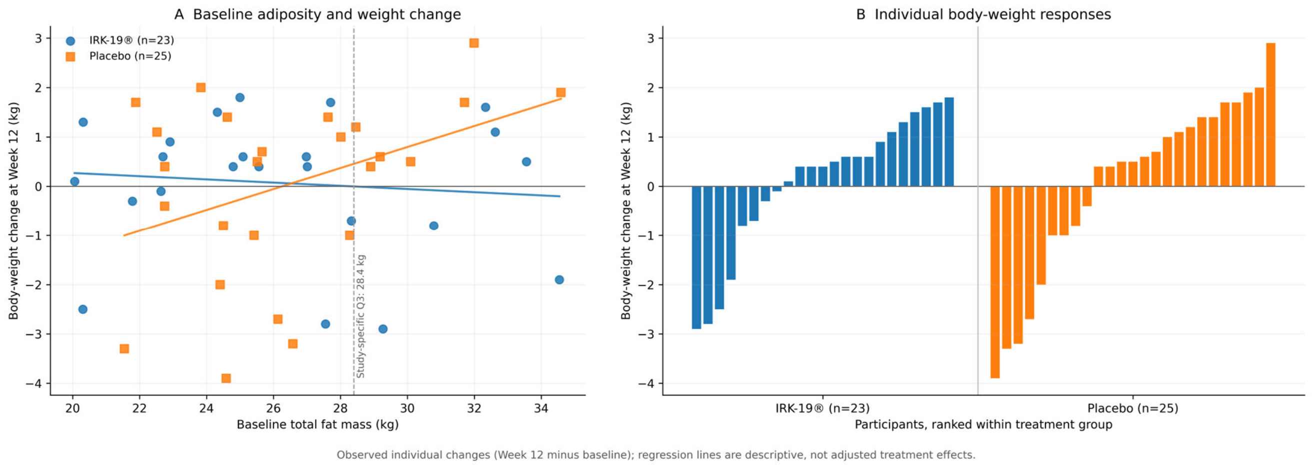

Figure S1. Individual body weight responses. Individual changes in body weight from baseline to week 12 are presented by randomized treatment group. The scatter plot shows the relationship between baseline DXA-derived total fat mass and individual body-weight change, with separate fitted regression lines for the IRK-19® and placebo groups. The individual-response plot illustrates the distribution and variability of body-weight changes within each group. Negative values indicate weight reduction from baseline. These plots are descriptive; the fitted lines do not establish a statistically significant treatment-by-baseline-fat-mass interaction. DXA, dual-energy X-ray absorptiometry.

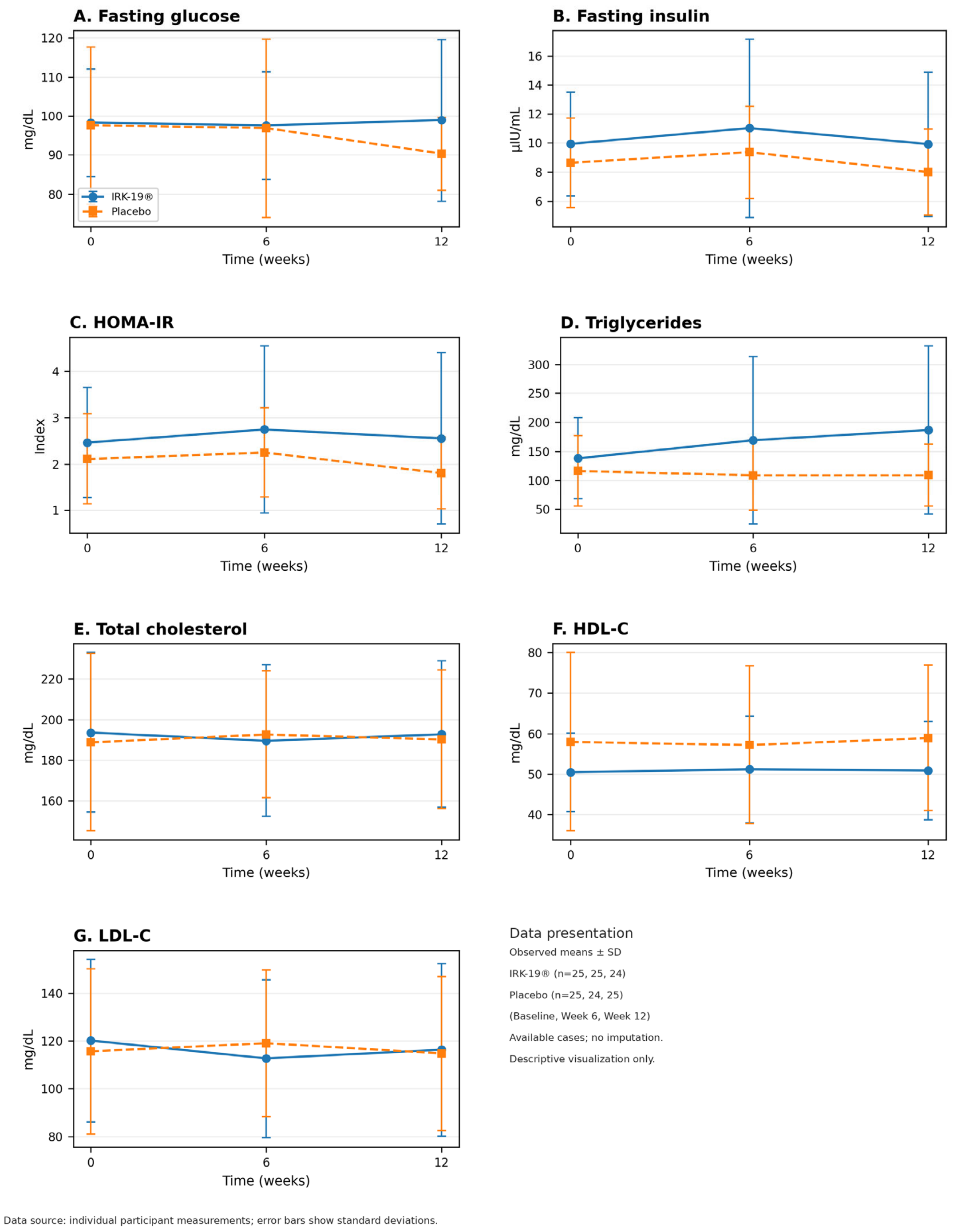

Figure S2. Longitudinal metabolic laboratory profiles during the 12-week intervention. Observed group means ± standard deviations (SDs) are shown at baseline, Week 6, and Week 12 for (A) fasting glucose, (B) fasting insulin, (C) homeostatic model assessment of insulin resistance (HOMA-IR), (D) triglycerides, (E) total cholesterol, (F) high-density lipoprotein cholesterol (HDL-C), and (G) low-density lipoprotein cholesterol (LDL-C). Blue solid lines with circular markers represent the IRK-19® group; orange dashed lines with square markers represent the placebo group. The numbers of participants with available measurements at baseline, Week 6, and Week 12 were 25, 25, and 24 for the IRK-19® group and 25, 24, and 25 for the placebo group, respectively. Error bars indicate SDs. These plots present observed, unadjusted group means; ANCOVA-adjusted between-group differences are reported separately in the manuscript. Missing values were not imputed.

A

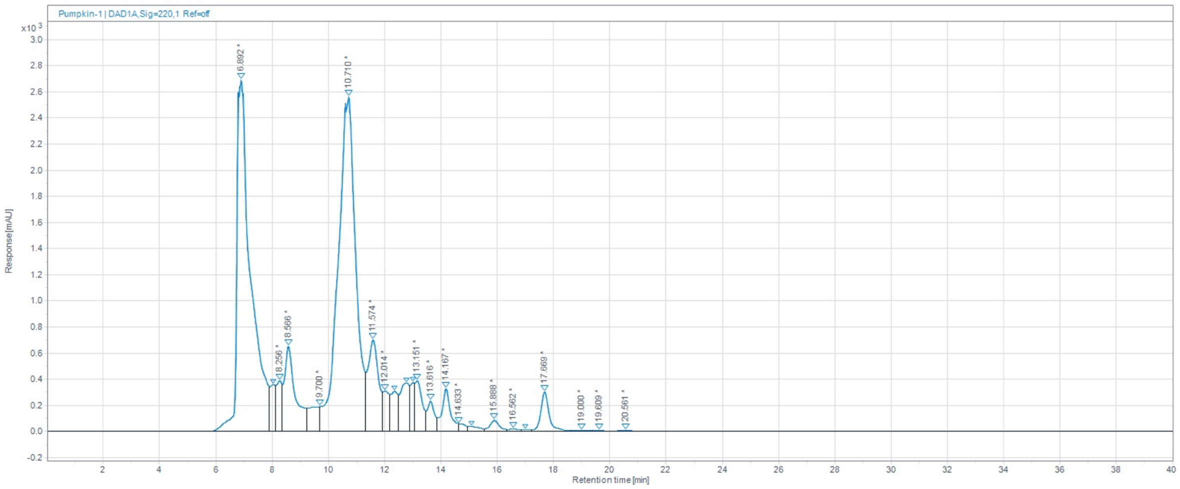

B Peak-integration data

Peak	Apparent MW interval (Da)	RT (min)	Area (mAU·s)	Area (%)	Start (min)	End (min)
1	≥29,473.5	6.892	92131.498	29.863	0.007	7.898
2		8.024	4600.367	1.491	7.898	8.115
3		8.256	5353.882	1.735	8.115	8.354
4		8.566	17840.560	5.783	8.354	9.221
5	21,896.2–29,473.5	9.700	5329.393	1.727	9.221	9.700
6	6,288.3–21,896.2	10.710	110447.312	35.800	9.700	11.313
7	3,747.1–6,288.3	11.574	18477.916	5.989	11.313	11.910
8	2,957.9–3,747.1	12.014	4857.900	1.575	11.910	12.179
9	2,273.8–2,957.9	12.340	5284.211	1.713	12.179	12.478
10	1,594.3–2,273.8	12.767	8375.928	2.715	12.478	12.885
11	1,373.8–1,594.3	12.997	3812.007	1.236	12.885	13.058
12	≤1,373.8	13.151	6789.650	2.201	13.058	13.454
13		13.616	4238.115	1.374	13.454	13.866
14		14.167	7123.543	2.309	13.866	14.632
15		14.633	939.952	0.305	14.632	14.936
16		15.090	1121.392	0.363	14.936	15.528
17		15.888	2143.435	0.695	15.528	16.347
18		16.562	514.540	0.167	16.347	16.849
19		16.990	315.528	0.102	16.849	17.232
20		17.669	6444.137	2.089	17.232	18.619
21		19.000	362.227	0.117	18.619	19.402
22		19.609	206.531	0.067	19.402	20.059
23		20.561	1804.534	0.585	20.059	40.000

Apparent MW ≤6.29 kDa (peaks 7–23): 23.602% of the total integrated UV area (reported as 23.6%).

SEC-UV area fractions describe relative chromatographic response, not absolute peptide content or purity.

MW interval boundaries correspond to retention times of 9.221 min (29,473.5 Da) and 13.058 min (1,373.8 Da).

Figure S3. SEC-UV chromatogram and peak-integration data of the IRK-19® clinical trial batch. (A) Representative size-exclusion chromatography with ultraviolet detection (SEC-UV) chromatogram of IRK-19® production batch 2205100101, recorded at 220 nm. (B) Corresponding peak-integration data showing retention time, apparent molecular-weight interval, integrated peak area, relative area percentage, and integration boundaries. Components with apparent molecular weights ≤6.29 kDa (peaks 7–23) accounted for 23.602% of the total integrated UV area (reported as 23.6%). Molecular-weight intervals were assigned using the calibration relationship between retention time and apparent molecular weight. The SEC-UV area percentage represents a relative chromatographic response and should not be interpreted as absolute peptide content, peptide purity, or sequence identification.

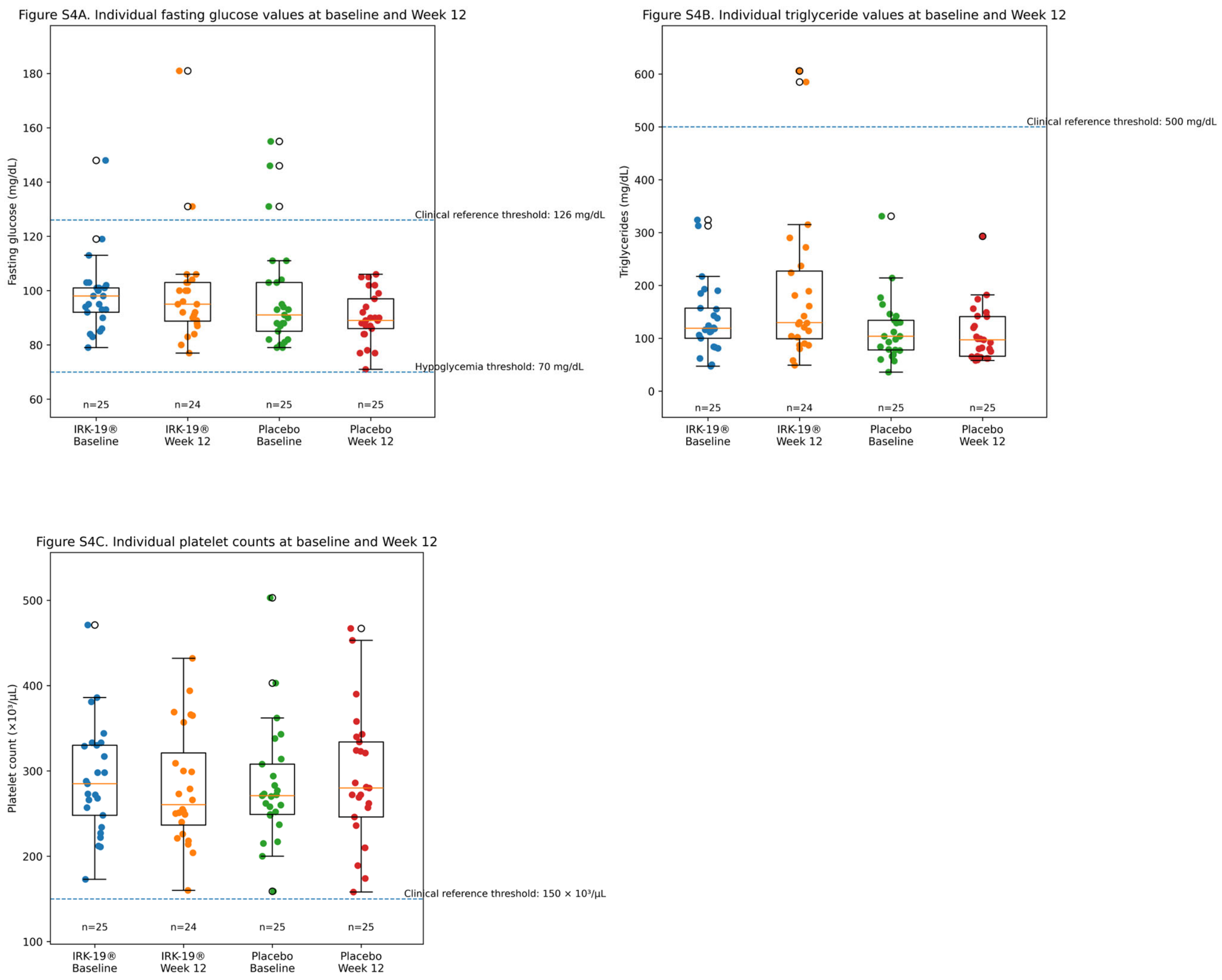

Figure S4. Individual fasting-glucose, triglyceride, and platelet-count measurements at baseline and Week 12. Individual measurements are shown for the IRK-19® and placebo groups: (A) fasting glucose (mg/dL), (B) triglycerides (mg/dL), and (C) platelet count (×10^3/µL). Colored points represent individual observations, and boxplots summarize the distributions at each assessment. Open circles indicate observations beyond the upper or lower boxplot whiskers. Dashed horizontal lines indicate clinical reference thresholds of 126 mg/dL for fasting glucose, 70 mg/dL for hypoglycemia, 500 mg/dL for triglycerides, and 150 × 10^3/µL for platelet count. At Week 12, fasting glucose ≥126 mg/dL and triglycerides ≥500 mg/dL were each observed in 2/24 participants in the IRK-19® group and 0/25 participants in the placebo group. No participant in either group had a Week 12 platelet count <150 × 10^3/µL. Sample sizes were 25 per group at baseline and 24 (IRK-19®) and 25 (placebo) at Week 12. The plots present observed individual measurements without covariate adjustment; ANCOVA-adjusted between-group estimates are reported separately in the manuscript.

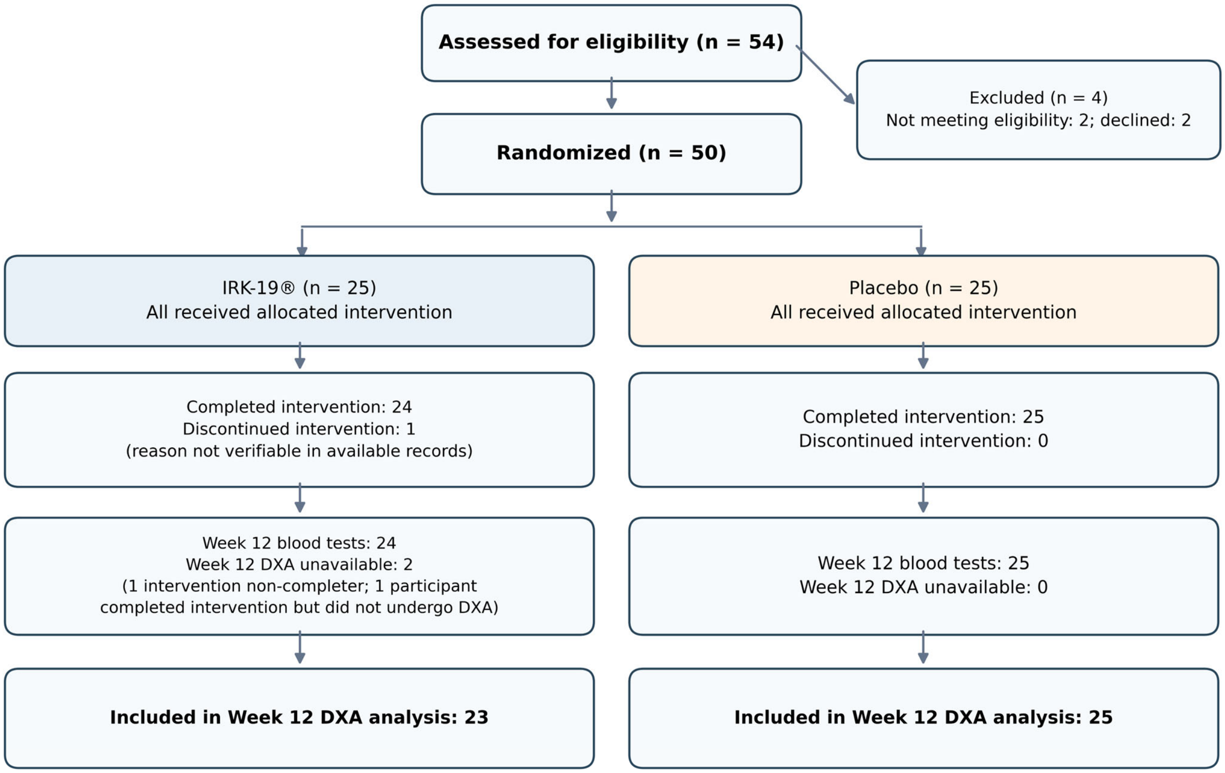

Total: 50 randomized; 49 completed intervention; 49 had Week 12 blood tests; 48 had Week 12 DXA.

DXA, dual-energy X-ray absorptiometry. The two participants without Week 12 DXA data were distinct individuals.

Based on the available study records and the CONSORT 2025 reporting framework.

Figure S5. Participant flow through the 12-week randomized, double-blind, placebo-controlled trial. Of 54 individuals assessed for eligibility, four were excluded (two did not meet the eligibility criteria and two declined participation). Fifty participants were randomized equally to the IRK-19® and placebo groups (n = 25 per group), and all received their allocated intervention. One participant in the IRK-19® group discontinued the intervention; 49 participants completed the 12-week intervention. At Week 12, laboratory data were available for 49 participants (IRK-19®, n = 24; placebo, n = 25), and evaluable dual-energy X-ray absorptiometry (DXA) data were available for 48 participants (IRK-19®, n = 23; placebo, n = 25). The two IRK-19® participants without Week 12 DXA data were distinct individuals: one discontinued the intervention, whereas the other completed the intervention but did not undergo the final DXA assessment. The reason for intervention discontinuation could not be verified from the available records. The flow diagram was prepared using the available study records and the CONSORT 2025 reporting framework.

Table S1. Summary of adverse events, participant disposition, and selected laboratory safety findings			
12-week randomized trial; randomized/treated population: IRK-19® n=25; placebo n=25.			
**Safety outcome**	**IRK-19®**	**Placebo**	**Total**
**A. Recorded clinical adverse events**			
Participants with ≥1 recorded AE, n (%)	0/25 (0.0%)	0/25 (0.0%)	0/50 (0.0%)
Participants with ≥1 recorded SAE, n (%)	0/25 (0.0%)	0/25 (0.0%)	0/50 (0.0%)
Discontinuations attributed to a recorded AE, n (%)			
Did not complete the intervention, n (%)	1/25 (4.0%)	0/25 (0.0%)	1/50 (2.0%)
Week 12 DXA result unavailable, n (%)	2/25 (8.0%)	0/25 (0.0%)	2/50 (4.0%)
**C. Selected Week 12 laboratory safety findings**			
Participants with available Week 12 laboratory results, n	24	25	49
Fasting glucose ≥126 mg/dL, n/N (%)	2/24 (8.3%)	0/25 (0.0%)	2/49 (4.1%)
Triglycerides ≥500 mg/dL, n/N (%)	2/24 (8.3%)	0/25 (0.0%)	2/49 (4.1%)
Platelet count <150 × 10^3/µL, n/N (%)	0/24 (0.0%)	0/25 (0.0%)	0/49 (0.0%)

Notes

AE = adverse event; SAE = serious adverse event; DXA = dual-energy X-ray absorptiometry. Recorded clinical events and measured laboratory abnormalities are distinct outcomes; no clinical AE/SAE was documented in the available study event records.

The IRK-19® group had one participant who did not complete the intervention; a separate participant completed intervention but declined the final DXA assessment. The reason for intervention discontinuation should not be assumed to be an AE.

Laboratory counts are based on participants with an available Week 12 measurement (IRK-19® n=24; placebo n=25). Thresholds were used for descriptive screening only and do not establish a clinical diagnosis or product causality.

No confirmatory tests, clinical adjudication, or subsequent follow-up for the elevated glucose/triglyceride values were available for this reanalysis. Serial liver and renal function data were unavailable; hepatic and renal safety cannot be assessed from these materials.

Table S2. Metabolic and hematological laboratory outcomes

A. Metabolic outcomes

Outcome	Unit	Baseline IRK-19® (n=25)	Baseline Placebo (n=25)	Week 6* IRK-19® (n=25)	Week 6* Placebo (n=24)	Week 12* IRK-19® (n=24)	Week 12* Placebo (n=25)	Week 12* adjusted difference (95% CI; ANCOVA n=49)	p value
**Fasting glucose**	mg/dL	98.24 ± 13.76	97.56 ± 20.09	97.52 ± 13.81	96.83 ± 22.82	98.88 ± 20.73	90.32 ± 9.35	+9.00 (1.93 to 16.07)	0.014
**Insulin**	μIU/mL	9.93 ± 3.57	8.63 ± 3.08	11.01 ± 6.14	9.36 ± 3.17	9.92 ± 4.97	7.99 ± 2.95	+0.95 (-0.79 to 2.70)	0.277
**HOMA-IR**	—	2.46 ± 1.19	2.11 ± 0.97	2.75 ± 1.80	2.25 ± 0.96	2.55 ± 1.85	1.81 ± 0.78	+0.44 (-0.08 to 0.96)	0.093
**Total cholesterol**	mg/dL	193.68 ± 39.12	188.88 ± 43.49	189.64 ± 37.22	192.75 ± 31.07	192.83 ± 35.86	190.32 ± 33.98	-2.13 (-18.16 to 13.90)	0.790
**Triglycerides**	mg/dL	138.00 ± 69.70	116.28 ± 60.92	169.24 ± 144.91	108.92 ± 60.92	186.67 ± 145.27	108.92 ± 53.70	+70.16 (9.90 to 130.41)	0.024
**HDL-C**	mg/dL	50.44 ± 9.73	58.00 ± 21.98	51.16 ± 13.21	57.25 ± 19.45	50.88 ± 12.18	58.96 ± 17.96	-2.25 (-5.94 to 1.43)	0.224
**LDL-C**	mg/dL	120.08 ± 34.07	115.56 ± 34.61	112.60 ± 33.09	118.96 ± 30.78	116.25 ± 36.12	114.72 ± 32.25	-4.04 (-18.35 to 10.28)	0.573

B. Hematological outcomes

Outcome	Unit	Baseline IRK-19® (n=25)	Baseline Placebo (n=25)	Week 6* IRK-19® (n=25)	Week 6* Placebo (n=24)	Week 12* IRK-19® (n=24)	Week 12* Placebo (n=25)	Week 12* adjusted difference (95% CI; ANCOVA n=49)	p value
**WBC**	10^3/μL	6.91 ± 1.89	6.53 ± 1.72	7.47 ± 1.52	6.22 ± 1.59	6.88 ± 1.40	6.57 ± 2.22	-0.08 (-0.93 to 0.77)	0.845
**RBC**	10^6/μL	4.61 ± 0.43	4.60 ± 0.49	4.66 ± 0.42	4.61 ± 0.50	4.68 ± 0.38	4.58 ± 0.45	+0.07 (-0.04 to 0.19)	0.207
**Hemoglobin**	g/dL	13.77 ± 1.32	13.52 ± 1.53	13.94 ± 1.38	13.46 ± 1.52	13.88 ± 1.37	13.30 ± 1.63	+0.32 (-0.05 to 0.68)	0.089
**Hematocrit**	%	40.56 ± 3.75	39.90 ± 4.24	41.06 ± 3.82	39.99 ± 4.46	40.97 ± 3.48	39.49 ± 4.61	+0.80 (-0.41 to 2.02)	0.190
**Platelet**	10^3/μL	290.24 ± 65.56	282.72 ± 69.59	289.00 ± 59.96	288.50 ± 73.13	281.25 ± 68.15	290.20 ± 77.69	-17.62 (-33.81 to -1.43)	0.034
**MCV**	fL	88.27 ± 6.06	86.75 ± 3.99	88.24 ± 5.52	86.78 ± 4.31	87.68 ± 5.74	86.20 ± 4.93	+0.38 (-0.70 to 1.46)	0.479
**MCH**	pg	29.97 ± 2.21	29.41 ± 1.64	29.96 ± 2.26	29.23 ± 1.72	29.64 ± 2.08	29.03 ± 1.87	+0.23 (-0.11 to 0.57)	0.185

**MCHC**	g/dL	33.92 ± 0.73	33.88 ± 0.59	33.93 ± 0.81	33.67 ± 0.62	33.81 ± 0.67	33.68 ± 0.63	+0.12 (-0.16 to 0.41)	0.389
**RDW-CV**	%	13.44 ± 1.23	13.54 ± 0.87	13.42 ± 1.20	13.51 ± 0.81	13.41 ± 1.00	13.50 ± 1.06	-0.06 (-0.44 to 0.32)	0.757
**Neutrophil**	%	58.88 ± 9.20	59.62 ± 6.97	62.24 ± 6.55	58.77 ± 6.66	58.40 ± 9.96	59.51 ± 7.13	-0.80 (-5.18 to 3.59)	0.716
**Lymphocyte**	%	30.00 ± 7.59	29.82 ± 6.23	27.84 ± 5.46	30.47 ± 6.18	30.25 ± 7.81	30.09 ± 6.41	+0.39 (-3.26 to 4.04)	0.831
**Monocyte**	%	7.48 ± 2.34	6.86 ± 1.55	6.51 ± 1.34	7.31 ± 2.11	7.39 ± 1.91	6.96 ± 1.64	+0.07 (-0.89 to 1.03)	0.886
**Eosinophil**	%	2.92 ± 1.83	2.97 ± 2.52	2.74 ± 1.88	2.76 ± 1.92	3.26 ± 2.96	2.75 ± 2.15	+0.47 (-0.53 to 1.47)	0.346
**Basophil**	%	0.71 ± 0.37	0.73 ± 0.34	0.68 ± 0.28	0.69 ± 0.16	0.69 ± 0.30	0.69 ± 0.32	-0.00 (-0.16 to 0.15)	0.960

Notes: Values are observed mean ± SD summaries derived from the uploaded raw dataset. Adjusted week-13 differences represent IRK-19® vs placebo estimates from ANCOVA models adjusted for the corresponding baseline value, age, and sex. Laboratory analyses are exploratory; p values are not adjusted for multiplicity. * The Excel columns W7 and W13 correspond to approximately 6 and 12 weeks of intervention, respectively. Each treatment-cell value is mean ± SD; n appears in the column heading.

Supplementary Table S3. Analytical characterization of IRK-19® production batches and six representative peptide markers.

A. Batch-specific compositional and analytical quality indices

Batch no.	Clinical trial batch	Crude protein (%)	SEC/GPC-UV ≤6.29 kDa (%)[1]	Six-marker total by HPLC (%)[2]
**2204110101**	No	67.2	23.0	2.63
**2205100101**	**Yes**	**66.2**	**23.6**	**2.79**
**2205260101**	No	65.3	23.2	2.65

B. Six mass-spectrometry-identified candidate peptide markers

Marker ID	Amino-acid sequence	Sequence identification	Quality-monitoring method
**P1**	GGEQQDQEGEEERE	Mass spectrometry[3]	HPLC using purified reference standards[3]
**P2**	GGEQQDQEGEEEREAEREY	Mass spectrometry[3]	HPLC using purified reference standards[3]
**P3**	SRGGEQQDQEGEEE	Mass spectrometry[3]	HPLC using purified reference standards[3]
**P4**	SRGGEQQDQEGEEER	Mass spectrometry[3]	HPLC using purified reference standards[3]
**P5**	SRGGEQQDQEGEEEREAEREY	Mass spectrometry[3]	HPLC using purified reference standards[3]
**P6**	NIIKVEGPL	Mass spectrometry[3]	HPLC using purified reference standards[3]

Notes. Values in Part A are reported in the respective certificates of analysis (COAs). Batch 2205100101 was used in the clinical trial.

[1] The SEC/GPC-UV result refers to the reported low-apparent-molecular-weight chromatographic fraction (≤6.29 kDa); it is an instrument- and calibration-dependent UV-area index, not an absolute peptide mass fraction, purity, or sequence identification.

[2] The HPLC result represents the combined content of the six marker peptides listed in Part B, not the total peptide content of the preparation. Individual peptide contents are not reported here.

[3] The sequence-identification and marker-monitoring workflows were confirmed by the product developer. Detailed mass-spectrometric identification records, individual marker quantification, and HPLC method-performance data are not included in the supplied COAs and should be cited or added only after source-record verification.

Source documents. COAs for IRK-19® batches 2204110101, 2205100101, and 2205260101; peptide sequences and analytical workflow provided by the product developer.
